# Protective effect of heme oxygenase induction in ethinylestradiol-induced cholestasis

**DOI:** 10.1111/jcmm.12401

**Published:** 2015-02-16

**Authors:** Lucie Muchova, Katerina Vanova, Jakub Suk, Stanislav Micuda, Eva Dolezelova, Leos Fuksa, Dalibor Cerny, Hassan Farghali, Miroslava Zelenkova, Martin Lenicek, Ronald J Wong, Hendrik J Vreman, Libor Vitek

**Affiliations:** a Institute of Medical Biochemistry and Laboratory Diagnostics, 1^st^ Faculty of Medicine, Charles University in PraguePrague, Czech Republic; b Department of Pharmacology, Charles University in Hradec KralovePrague, Czech Republic; c Department of Biological and Medical Sciences, Faculty of Pharmacy, Charles University in PraguePrague, Czech Republic; d Department of Pharmacology, 1^st^ Faculty of Medicine, Charles University in PraguePrague, Czech Republic; e Department of Pediatrics, Stanford University School of MedicineStanford, CA, USA; f 4^th^ Department of Internal Medicine, 1^st^ Faculty of Medicine, Charles University and General Faculty Hospital in PraguePrague, Czech Republic

**Keywords:** 17α- ethinylestradiol, heme, nuclear factor erythroid-2-related factor-2, bile acids, multidrug resistance-associated protein 3

## Abstract

Estrogen-induced cholestasis is characterized by impaired hepatic uptake and biliary bile acids secretion because of changes in hepatocyte transporter expression. The induction of heme oxygenase-1 (HMOX1), the inducible isozyme in heme catabolism, is mediated *via* the Bach1/Nrf2 pathway, and protects livers from toxic, oxidative and inflammatory insults. However, its role in cholestasis remains unknown. Here, we investigated the effects of HMOX1 induction by heme on ethinylestradiol-induced cholestasis and possible underlying mechanisms. Wistar rats were given ethinylestradiol (5 mg/kg s.c.) for 5 days. HMOX1 was induced by heme (15 μmol/kg i.p.) 24 hrs prior to ethinylestradiol. Serum cholestatic markers, hepatocyte and renal membrane transporter expression, and biliary and urinary bile acids excretion were quantified. Ethinylestradiol significantly increased cholestatic markers (*P* ≤ 0.01), decreased biliary bile acid excretion (39%, *P* = 0.01), down-regulated hepatocyte transporters (Ntcp/Oatp1b2/Oatp1a4/Mrp2, *P* ≤ 0.05), and up-regulated Mrp3 (348%, *P* ≤ 0.05). Heme pre-treatment normalized cholestatic markers, increased biliary bile acid excretion (167%, *P* ≤ 0.05) and up-regulated hepatocyte transporter expression. Moreover, heme induced Mrp3 expression in control (319%, *P* ≤ 0.05) and ethinylestradiol-treated rats (512%, *P* ≤ 0.05). In primary rat hepatocytes, Nrf2 silencing completely abolished heme-induced Mrp3 expression. Additionally, heme significantly increased urinary bile acid clearance *via* up-regulation (Mrp2/Mrp4) or down-regulation (Mrp3) of renal transporters (*P* ≤ 0.05). We conclude that HMOX1 induction by heme increases hepatocyte transporter expression, subsequently stimulating bile flow in cholestasis. Also, heme stimulates hepatic Mrp3 expression *via* a Nrf2-dependent mechanism. Bile acids transported by Mrp3 to the plasma are highly cleared into the urine, resulting in normal plasma bile acid levels. Thus, HMOX1 induction may be a potential therapeutic strategy for the treatment of ethinylestradiol-induced cholestasis.

## Introduction

Estrogens are known to cause intrahepatic cholestasis in susceptible women during pregnancy, administration of oral contraceptives or hormone replacement therapy 1. In fact, it is a rather frequent condition, with a prevalence rate of intrahepatic cholestasis of pregnancy reaching 0.2–1.5% in Europe and USA 2. Induction of cholestasis by the synthetic estrogen, 17α-ethinylestradiol (EE), has been used as an experimental model of human intrahepatic cholestasis 3.

The mechanisms involved in EE-induced cholestasis are multifactorial and include reduction in both bile salt-dependent 4 as well as independent 5 bile flow and the subsequent increase of serum bile acids (BA). Functional analyses revealed diminished sinusoidal uptake and canalicular transport of BA caused by down-regulation of the main membrane transporters – sinusoidal NTCP (Na^+^-taurocholate co-transporting polypeptide, *SLC10A1*) and OATPs (organic anion-transporting polypeptides, encoded by *SLCOs*) 3,6; and canalicular MRP2 (multidrug resistance-associated protein 2, encoded by *ABCC2*) and BSEP (bile salt export pump, encoded by *ABCB11*) 7,8. Estrogens are also implicated in reduced bile salt synthesis 9, increased tight junctions permeability 10, decreased plasma membrane fluidity and redistributed gangliosides within hepatocyte membranes 11,12.

Heme oxygenase (HMOX) is the rate-limiting enzyme in the heme catabolic pathway. It catalyses the degradation of heme to produce equimolar amounts of carbon monoxide (CO), iron and biliverdin, the latter being rapidly metabolized to bilirubin by biliverdin reductase 13. There are two structurally related HMOX isozymes, the inducible HMOX1 (OMIM*141250), also called heat-shock protein 32 (HSP32), and the constitutive HMOX2 (OMIM *141251) 14. The induction of HMOX1 by its substrate, heme, is mediated *via* Bach1/Nrf2 (nuclear factor erythroid-2-related factor-2) pathway 15. Over the past decade, enhanced HMOX enzymatic activity has emerged as an important mediator of antioxidant, cytoprotective, neurotransmitter and anti-inflammatory actions mediated by the production of its bioactive products, CO and bilirubin 16–18. Moreover, a number of animal as well as clinical studies emphasize the crucial role of HMOX in the protection against oxidative stress-mediated diseases including atherosclerosis 19, diabetes 20, hypertension 21 and cancer 22.

In the liver, the HMOX1 and HMOX2 isozymes have distinct topographic patterns. HMOX1 is expressed predominantly in Kupffer cells, while the constitutive HMOX2 is abundant in hepatocytes 23. Suemetsu *et al*. 24 have shown that CO derived from HMOX2 is necessary to maintain liver sinusoids in a relaxed state, and this process is mediated by mechanisms involving soluble guanylate cyclase in hepatic stellate cells. *In vivo*, HMOX1 induction has been shown to protect mice and rats from apoptotic liver damage because of liver graft rejection as well as from ischaemia/reperfusion injury 25,26. Furthermore, CO contributes to the maintenance of blood perfusion in the liver and to the excretion of bile 27. In another study, stress-induced levels of CO (up to concentrations of 4–5 μmol/L) were shown to stimulate bile secretion in a dose-dependent manner, although further administration of higher amounts of CO caused a reduction of bile output by mechanisms appearing to involve hepatocyte membrane transporter Mrp2 28. In addition, CO has been shown to limit the contractility of bile canaliculi by suppressing intracellular calcium mobilization 29 and modulate the expression of liver transporters 30,31. Also, retention of bilirubin, a potent antioxidant product of heme catabolic pathway, might play an important cytoprotective role in cholestasis as well 32.

The objective of this study was to investigate whether induction of HMOX by heme prevents EE-induced cholestasis in rats and to identify the possible underlying mechanisms.

## Material and methods

### Chemicals

EE, NADPH, hemin, sulfosalicylic acid (SSA), bilirubin, bovine serum albumin, taurocholic acid, glutathione, glutathione-reductase, 5,5′-dithio-bis(2-nitrobenzoic acid) (DTNB) were purchased from Sigma-Aldrich (St. Louis, MO, USA).

### Reagents

#### Potassium phosphate buffer, 0.1 M, pH 7.4

Dihydrogen potassium phosphate, 13.61 g, was dissolved in distilled water. The pH was adjusted to 7.4 with KOH (0.1 M). The final volume was brought to 1 l with distilled water 33.

#### Methemalbumin, 0.15 mM

Hemin, 9.9 mg, was dissolved in 2.5 ml of 0.4 M Na_3_PO_4_. Distilled water was added to a volume of 8 ml and 100 mg of bovine serum albumin was dissolved. The pH was gradually adjusted to 7.4 by using 1.0 N HCl with vigorous stirring. Distilled water was added to bring the total volume to 10 ml 33. Heme was always administered in the form of methemalbumin to animals or used for *in vitro* experiments.

### Animals

Adult female Wistar rats obtained from Anlab (Prague, Czech Republic) weighing 200–280 g, were provided with water and food *ad libitum*. All aspects of the animal studies met the accepted criteria for the care and experimental use of laboratory animals. All protocols were approved by the Animal Research Committee of the 1st Faculty of Medicine, Charles University in Prague.

Rats were randomly divided into 4 groups: (1) those receiving only vehicle (propanediol) or CON; (2) those administered 5 mg/kg EE daily i.p. for 5 consecutive days (days 1–5) or EE; (3) those receiving 15 μmol/kg heme i.p. on days 0 and 3) or HC; and (4) those co-administered heme and EE at the above-mentioned doses or HE. Each group included at least 8 animals.

Surgical procedures were performed on day 6 between 8 and 10 a.m. Experimental animals were anaesthetized with sodium pentobarbital (50 mg/kg i.p.). Biliary trees were then exposed through midline abdominal incisions. Bile ducts were cannulated and bile was collected for 20 min. (equilibration) and then in two 30-min. intervals (20–50 and 50–80). In addition, all rats were cannulated with polyethylene tubes in the left carotid artery for blood sampling and urinary bladder for urine collection. Urine was collected in three sessions: first one for 20 min. (equilibration) and then in two 30-min. intervals (20–50 and 50–80). For biliary and urinary BA output, the 20–50 collections of bile and urine were used. Body temperature was maintained at 37°C by using a heated platform. At the end of each experiment, animals were killed by exsanguination, and the livers were removed and weighed.

### Markers of cholestasis

Serum biochemical markers [alkaline phosphatase (ALP), alanine aminotransferase (ALT), bilirubin] were determined in an automatic analyser (Modular analyser; Roche Diagnostics GmbH, Mannheim, Germany) by using standard assays. Total serum and biliary BA levels were determined spectrophotometrically by using a Bile Acids kit (Trinity Biotech, Jamestown, NY, USA). BA levels in urine were determined by gas chromatographic/mass spectrophotometric method as previously described 34.

### Total HMOX enzyme activity determinations

Twenty microlitres of 10% liver sonicate [2 mg fresh weight (FW)] was incubated for 15 min. at 37°C in CO-free septum-sealed vials containing identical volumes of 150 μM heme and 4.5 mM NADPH as previously described 33. Blank reaction vials contained potassium phosphate buffer in place of NADPH. Reactions were terminated by adding 5 μl of 30% (w/v) SSA. The amount of CO generated by the reaction and released into the vial headspace was quantified by gas chromatography with a Reduction Gas Analyser (Peak Laboratories, Mountain View, CA, USA). HMOX activity was calculated as pmol CO/h/mg FW.

### HMOX-1 mRNA determinations

Total liver RNA was isolated by using TRIzol reagent (Invitrogen, Carlsbad, CA, USA) and cDNA was generated by using an iScript reverse transcription kit (Bio-Rad Laboratories, Hercules, CA, USA). Real-time PCR was performed with TaqMan® Gene Expression Assay Kit for following genes: *Ntcp* (*Slc10a1*, Rn00566894_m1), *Oatp1a1* (*Slco1a1*, Rn00755148_m1), *Oatp1a4* (*Slco1a4*, Rn00756233_m1), *Bsep* (*Abcb11*, Rn00582179_m1), *Mrp2* (*Abcc2*, Rn00563231_m1), *Mrp3* (*Abcc3*, Rn01452854_m1), *Mrp4* (*Abcc4,* Rn01465702_m1), *Hmox1* (*Hmox1*, Rn00561387_m1), *Asbt* (*Slc10a2*, Rn00691576_m1), *Osta* (*Ostalpha*, Rn01763289_m1) and *Gapdh* rat endogenous control kit, all provided by Life Technologies (Carlsbad, CA, USA).

### Biliary total glutathione determinations

Glutathione was determined in a bile sample collected for 20 min. Bile was mixed with five volumes of SSA (5% w/v in distilled water) and stored at −80°C until analysis. Total glutathione was measured as previously described 35. Briefly, bile samples were first diluted 500-fold by using a phosphate (100 mM)/EDTA (1 mM) buffer (pH 7.4). Diluted bile samples (50 μl) were transferred to 96-well microplate and mixed with 100 μl of recycling agent (containing 0.30 mM NADPH, 0.225 mM DTNB and 1.6 U/ml glutathione-reductase in an EDTA phosphate buffer). Immediately after recycling agent addition, colour development was recorded at 405 nm for 4 min. by using Tecan Sunrise™ microplate reader equipped with kinetic analysis software (Tecan group Ltd., Mannedorf, Switzerland).

### Primary rat hepatocyte culture and transient transfection assay

Primary hepatocytes were isolated from anaesthetized Wistar rats by the two-step collagenase perfusion as previously described 36. Hepatocytes with cell viability greater than 90% (as assessed by trypan blue staining) were first plated on 35-mm collagen-coated cell culture dishes and maintained at 37°C, 5% CO_2_ in William's medium E, supplemented with penicillin/streptomycin, L-glutamine, insulin and 10% foetal bovine serum. On the next day, Nrf2 gene was silenced with siRNA (Sigma-Aldrich) by using Lipofectamine RNAiMAX (Invitrogen) according to the manufacturer's instructions. The knockdown level of Nrf2 gene was verified by qRT-PCR and was always higher than 75%. Cells were treated with vehicle, TCA (10, 50, 100 μM), unconjugated bilirubin (25, 250 μM), EE (10 μM) and/or MHA (30 μM) 24 hrs after transfection.

### Statistical analyses

Normally distributed data are presented as means ± SD and analysed by Student's *t*-test and one-way anova with post-hoc Holm–Sidak test for multiple comparisons. Non-normally distributed data sets are expressed as medians (25%–75%) and analysed by Mann–Whitney rank sum test and nonparametric Kruskal–Wallis anova with Dunn′s correction. Data with highly skewed distributions were log-transformed. Differences were deemed statistically significant when *P* < 0.05.

## Results

### Induction of HMOX1 normalizes serum BA in cholestatic rats

As expected, compared with controls, induction of cholestasis with EE resulted in significant increases of total BA, ALP activity as well as total serum bilirubin levels (Table1). Heme pre-treatment resulted in normalization of total BA concentrations as well as ALP activity in cholestatic animals, while total bilirubin levels remained elevated. Application of heme to control animals had no effect on BA and ALP (cholestatic parameters), but total bilirubin levels significantly increased (most likely as a result of bilirubin formation from heme administered to this experimental group). No significant changes have been observed in the serum ALT activity, a marker of hepatocellular liver injury (data not shown).

**Table 1:** The effect of heme pre-treatment on serum cholestatic markers

Groups	Total serum bile acids (μmol/L)	ALP (μkat/L)	Total serum bilirubin (μmol/L)
Vehicle (CON)	26.0 ± 11.9	2.1 ± 0.8	2.0 ± 0.8
EE-treated (EE)	**54.3** ± 22.2*	**3.6** ± 1.1*	**5.2** ± 1.2*
Vehicle + heme (HC)	29.4 ± 17.4	2.2 ± 0.5	**5.1** ± 2.2*
EE + heme (HE)	**23.3** ± **17.2**†	**2.6** ± **1.3**†	**5.5** ± 1.4*

Bile acid (BA) concentrations, alkaline phosphatase (ALP) activity and total bilirubin levels were measured in sera of control (CON), ethinylestradiol (EE), heme (HC), and heme + EE (HE)-treated animals.

* *P* ≤ 0.05 *versus CON*

† *P* ≤ 0.05 *versus EE*.

### Total liver HMOX activity in EE-induced cholestasis

No significant differences in HMOX activity were observed in the livers of CON and EE groups (211 ± 22 *versus* 176 ± 27 pmolCO/h/mg FW, respectively, *P* = 0.85). As expected, heme pre-treatment resulted in an increase in liver HMOX activity in both HC and HE groups (353 ± 166 and 290 ± 52 pmolCO/h/mg FW, respectively, *P* ≤ 0.05) as compared with CON and EE groups.

### Cross-talk between EE and BA in primary rat hepatocytes

**Figure 1:**
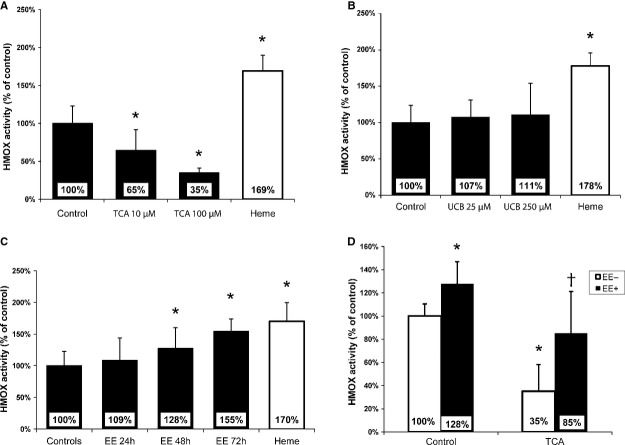
Total heme oxygenase (HMOX) activity in primary rat hepatocytes. Primary hepatocytes were incubated with: (A) taurocholic acid (TCA), (B) unconjugated bilirubin (UCB) for 24 hrs or with (C) 10 μM ethinylestradiol (EE) for 24, 48 and 72 hrs. (D) 10 μM EE was added to media 24 hrs before TCA treatment (100 μM, another 24 hrs). *P ≤ 0.05 *versus CON*, ^†^*P* ≤ 0.05 *versus TCA*. Hepatocytes treated with heme served as positive controls.

Because of the inhibitory effect of BA on HMOX activity in HepG2 cells *in vitro* and in obstructive cholestasis *in vivo* described previously by our group 32, we decided to investigate whether BA, EE, and bilirubin affected total HMOX enzyme activity in primary rat hepatocytes. We found that HMOX activity significantly decreased to 65% and 35% of CON levels 24 hrs after incubation with 10 or 100 μM taurocholic acid, respectively (*P* < 0.01) (Fig.1A). Bilirubin, ranging from 25 to 250 μM, had no effect on HMOX activity (Fig.1B). Similarly, incubation with 10 μM EE for 24 hrs had no effect on HMOX activity. However, significant increases were found 48 and 72 hrs (135% and 155%, *P* < 0.05, respectively) after treatment with 10 μM EE (Fig.1C). Moreover, EE treatment significantly diminished the inhibitory effect of taurocholic acid on HMOX activity (Fig.1D).

### Induction of HMOX1 increases biliary secretion of BA and glutathione in cholestatic rats

To identify the role of heme pre-treatment on bile production, we measured bile flow and biliary bile acids secretion rate in control and cholestatic animals with and without heme treatment (*n* = 6 in each group). A significant drop of bile flow to 24% (*P* < 0.001) was observed in EE-treated animals compared with controls. Importantly, heme pre-treatment of cholestatic animals resulted in slight increase of bile flow to 150% (*P* = 0.03) compared with those treated with EE. Administration of heme to control animals had no effect on bile flow (96%, *P* = 0.38).

In addition, biliary BA secretion decreased in cholestatic animals (EE) to 39% of CON values, but significantly increased after heme pre-treatment (HE 166% compared to EE, *P* = 0.04) (Table2).

**Table 2:** Effect of heme oxygenase (HMOX) induction on bile salt-dependent and -independent bile flow

Group of animals	Biliary bile acids output (μmol/g/min.)	Bile flow (μl/g/min.)	Biliary glutathione output (nmol/g/min.)
Vehicle (CON)	210.02 ± 43.59	2.21 ± 0.25	7.43 ± 2.34
EE-treated (EE)	81.64 ± 8.94*	0.54 ± 0.10*	0.32 ± 0.04*
Vehicle + heme (HC)	207.24 ± 12.03	2.13 ± 0.47	9.38 ± 0.17
EE + heme (HE)	135.52 ± 45.07*†	0.81 ± 0.25*†	0.52 ± 0.05*†

Bile volume, bile acids and glutathione concentrations were measured in the bile collected for 30 min. from control (CON), ethinylestradiol (EE), heme (HC) or heme + ethinylestradiol (HE)-treated animals and recalculated to grams of liver tissue.

* *P* ≤ 0.05 *versus CON*

† *P* ≤ 0.05 *versus EE*.

To clarify the effect of EE and heme on bile salt-independent bile flow in our experimental settings, we measured the biliary glutathione output in CON and cholestatic rats with or without heme pre-treatment. Compared with CON, biliary glutathione output was significantly reduced in EE-treated animals (to 4%, *P* = 0.03). Administration of heme to EE-treated animals led to an apparent increase (to 162%, *P* = 0.006, *versus* EE) in glutathione output, although the values did not reach CON values. Administration of heme to CON animals had no significant effect on glutathione output (126%, *P* = 0.18) (Table2).

### Effect of heme on expression of hepatocyte transporters

**Figure 2:**
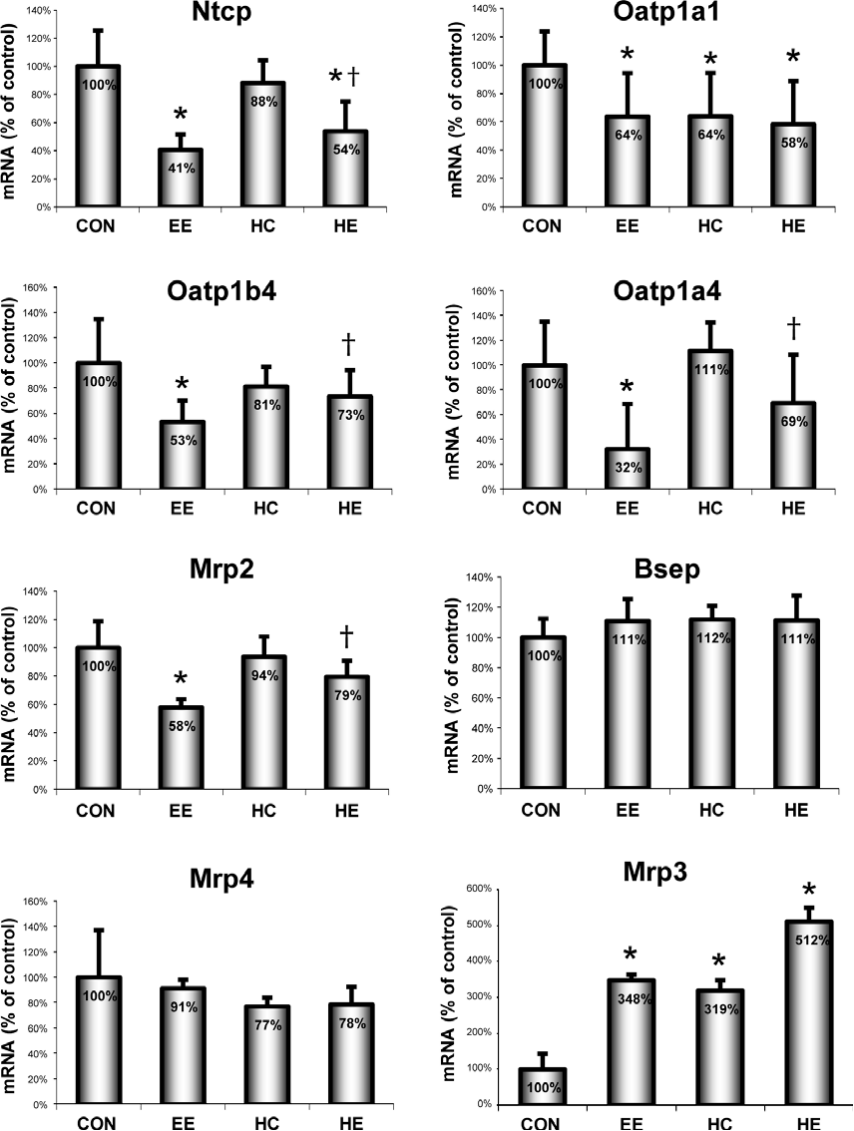
mRNA expression of key hepatic transporters. Relative expression of key sinusoidal (*Ntcp, Oatps, Mrp3* and *Mrp4)* and canalicular (*Mrp2, Bsep*) bile acid (BA) transporters was measured in the livers of control (CON), ethinylestradiol (EE), heme (HC) or heme + EE (HE)-treated animals. *P ≤ 0.05 *versus CON*, ^†^*P* ≤ 0.05 *versus EE*.

To elucidate the mechanism by which heme stimulates bile flow in EE-treated cholestasis, we measured the expression of key hepatocyte bile pigment and lipid transporters in the rat livers. EE treatment significantly decreased expression of sinusoidal *Ntcp* and *Oatps* (*Oatp1a1*, *Oatp1a4*, *Oatp1b2*) as well as canalicular *Mrp2* transporters. No effect was observed on the expression of sinusoidal *Mrp4* and canalicular *Bsep*. Importantly, heme pre-treatment of EE-exposed rats significantly increased mRNA of key hepatocyte transporters (*Ntcp*, *Oatp 1a4*, *Oatp1b2*, *Mrp2*; Fig.2). Interestingly, EE as well as heme, up-regulated sinusoidal *Mrp3* expression in CON rats (348% and 319%, respectively, *P* < 0.05). This increase was even more pronounced in cholestatic rats treated with heme (HE) (512%, *P* < 0.05).

**Figure 3:**
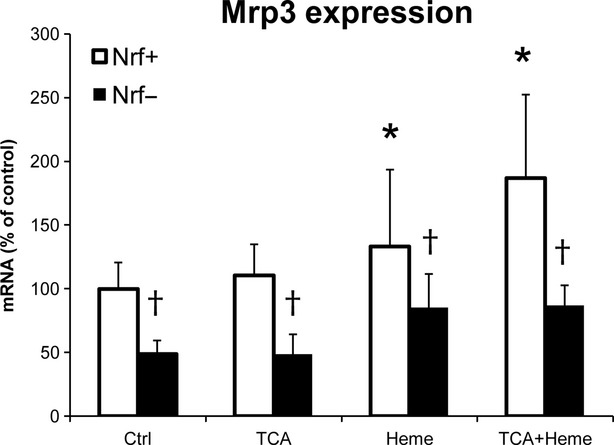
Mrp3 expression in primary rat hepatocytes. Relative expression of *Mrp3* transporter mRNA was measured in primary hepatocytes with (Nrf−) or without (Nrf+) Nrf2 silencing. Cells were treated with 50 μM taurocholic acid (TCA), 30 μM heme or both for 4 hrs. *P ≤ 0.05 *versus CON*, ^†^*P* ≤ 0.05 *versus corresponding Nrf+ group*.

In another set of experiments, we focused on mechanism of heme-induced *Mrp3* overexpression. We examined the effect of the main heme-activated transcription factor *Nrf2* on *Mrp3* expression in primary rat hepatocytes (Fig.3). While treatment of cells with heme or heme + taurocholic acid markedly increased the *Mrp3* expression, the silencing of *Nrf2* led to a significant decrease in *Mrp3* expression in all experimental groups.

### Induction of HMOX1 increases urinary BA output

**Figure 4:**
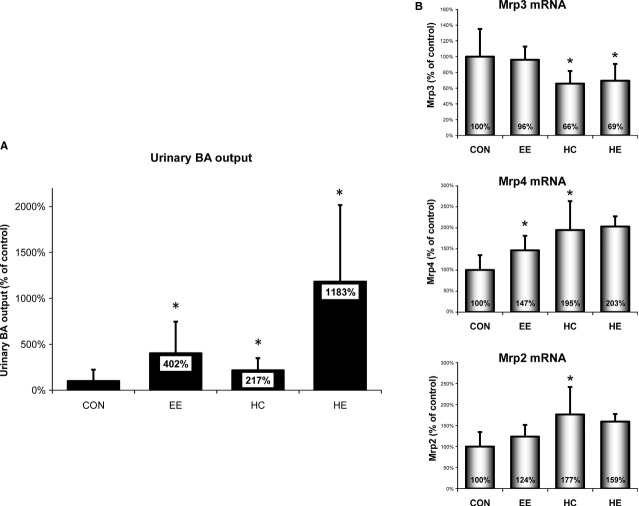
Urinary bile acid (BA) output and mRNA expression of renal BA transporters. (A) BA concentration was measured in the urine collected for 30 min. from control (CON), ethinylestradiol (EE), heme (HC), or heme + EE (HE)-treated animals and expressed as relative changes from CON. (B) Relative expression of key renal BA transporters was measured in kidneys of CON, EE, HC, or HE-treated animals. *P ≤ 0.05 *versus CON*.

The significant increases in *Mrp3* expressions in cholestasis as well as after heme pre-treatment (resulting in an increased transport of conjugated BA from hepatocytes to the bloodstream) together with low serum concentration of BA in HE rats prompted us to measure the extent of urinary BA output. As expected, urinary BA output significantly increased (402%) in cholestatic animals compared with CON (*P* = 0.04). Interestingly, administration of heme to CON animals resulted in a significant increase (217%) in urinary BA output and was even more pronounced in heme-pre-treated cholestatic rats when compared with CON (1183%, *P* < 0.05; Fig.4A).

Unlike in the liver, the expression of *Mrp3*, a renal BA reabsorption transporter, was significantly decreased following heme administration. On the other hand, heme caused significant increases in the expression of kidney *Mrp2* and *Mrp4*, important renal BA exporters (Fig.4B). We did not observe any significant changes in the expression levels of *Asbt* and *Osta* (data not shown).

## Discussion

In this study, we demonstrated that the induction of HMOX1 with its substrate, heme, can confer protection against EE-induced cholestasis by increasing both liver and renal clearance of BA in a rat model.

Recently, our group has shown that BA down-regulates HMOX activity *in vitro* and *in vivo* 32, and that this effect might be responsible for increased oxidative stress and subsequent liver injury in obstructive cholestasis. However, we did not observe any changes in HMOX activity in the livers of cholestatic EE-treated animals compared with controls. Parallel *in vitro* experiments using primary rat hepatocytes revealed opposite effects of BA and EE on HMOX activity. While taurocholic acid was found to be a potent HMOX inhibitor, prolonged treatment with EE resulted in significant increases in HMOX activity. Thus, we speculated that the observed unaffected HMOX activity following EE-induced cholestasis might be as a result of an interaction of the opposing effects of BA and estrogens.

Hepatoprotective effect of HMOX has been described earlier in an ischaemia–reperfusion injury, graft-versus*-*host reaction or sepsis 25,26,37. The hepatoprotection is believed to be conferred *via* HMOX metabolic products CO and biliverdin/bilirubin. While bilirubin protects the liver from oxidative stress triggered by high concentrations of BA 32, CO might have an effect on bile flow 24,27,28. Heme has been long considered strong pro-oxidant with harmful effect on various organ systems. However, its ability to induce HMOX1 and form biologically active products has recently been implicated in beneficial effects in various experimental models including inflammatory bowel disease 38, diabetes 39, non-alcoholic liver disease 40, arterial hypertension 41 or sepsis 42. In this study, we induced HMOX1 with heme applied in the form of methemalbumin to diminish the toxicity of free heme and increase formation of HMOX products.

In EE-treated animals, HMOX1 induction had clearly anti-cholestatic effect as measured by serum cholestatic markers. To understand this protective mechanism, we measured bile flow as well as biliary output of BA and glutathione, markers of bile salt-dependent and -independent bile flow, respectively. All these parameters were significantly increased in the heme pre-treated cholestatic group as compared with cholestasis without heme pre-treatment, although still much lower than in control group. A very similar pattern has been observed in the expression of key hepatic sinusoidal (*Oatps*, *Ntcp*) and canalicular (*Mrp2*) transporters, which were transcriptionally repressed in EE-treated, but to a much lesser extent in the heme pre-treated cholestatic groups. Recently, we have shown that inhaled CO can affect the expression of hepatic transporters 30, suggesting that CO generated in the HMOX pathway can contribute to an increase in the expression of liver transporters. It is important to note that the administration of heme resulted in an increase in total serum bilirubin levels in both control and cholestatic groups. In this case, we cannot consider bilirubin a cholestatic marker as its elevation in the serum was probably because of an increased formation arising heme degradation rather than the impaired clearance. Taken together, it appears that HMOX1 activation by heme can increase, but not normalize, both bile salt-dependent and -independent bile flow in cholestatic livers.

The only transporter specifically activated by heme was Mrp3. As described previously, Mrp3 is considered one of the basolateral overflow pumps compensating for impaired canalicular Mrp2 8,43. Interestingly, HMOX1 activation by heme is mediated *via* Nrf2, a transcriptional factor responsible for activation of many antioxidative stress genes 44. Moreover, transcriptional regulation of *Mrp3* by Nrf2 45, and a possible anti-cholestatic effect of this pathway in mice 46, has been suggested recently. In our study, we observed a significant increase in *Mrp3* in all heme and/or cholestatic groups with the highest increase when both cholestasis and heme were present (HE group). Parallel *in vitro* experiments with primary rat hepatocytes confirmed key role of Nrf2 in heme-mediated Mrp3 overexpression. Heme pre-treatment with/without TCA increased *Mrp3* expression, while Nrf2 silencing repressed both basal and stimulated expressions of *Mrp3*.

**Figure 5:**
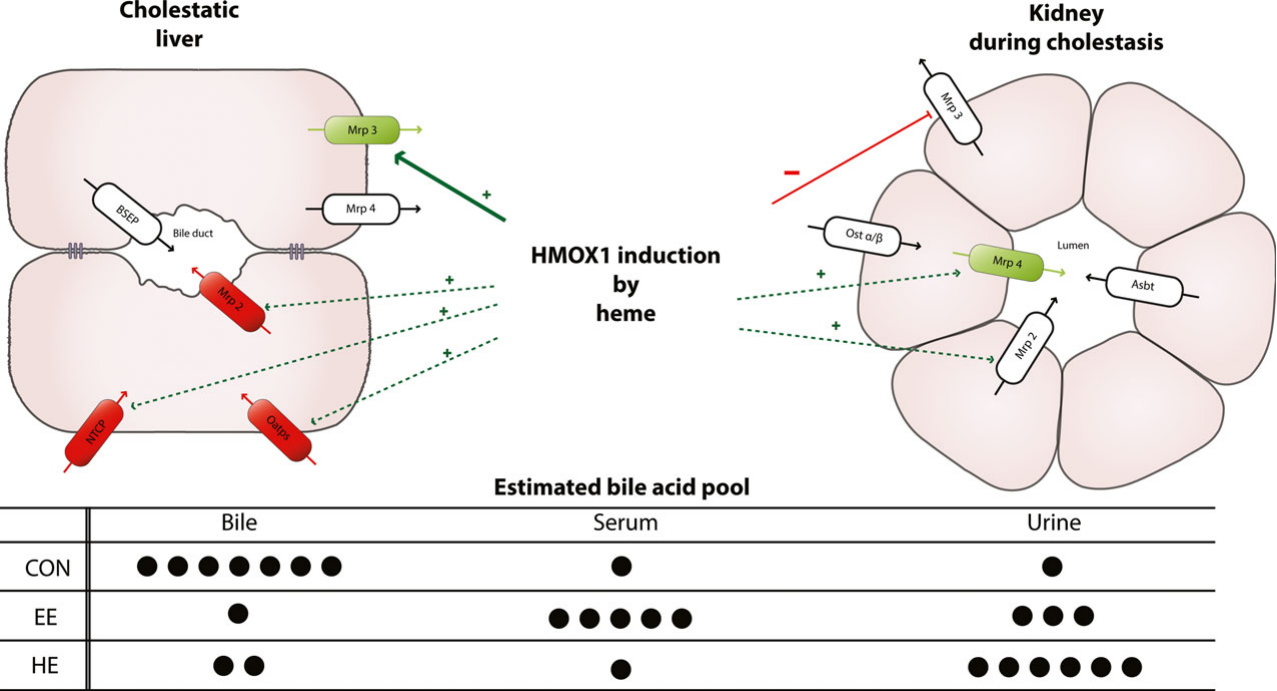
Proposed mechanism of heme oxygenase-1 (HMOX1) induction on liver and kidney transporters in EE-induced cholestasis. EE-induced cholestasis is characterized by either decrease (red ovals) or increase (green ovals) in the expression of key hepatocyte and kidney transporters. Induction of HMOX1 with heme increased (green arrows), brought close to CON values (dashed green arrows) or decreased (red arrows) expression of these transporters. The changes in the expression of liver and kidney transporters result in the redistribution of bile acid pool in cholestatic (EE) and heme pre-treated (HE) animals.

Despite high *Mrp3* levels, we observed normal plasma BA concentrations in both heme-treated groups (HC and HE). The fact that Mrp3 transports BA from hepatocytes to plasma for renal excretion prompted us to focus on renal clearance of BA. As expected, we found an increase in urinary BA output in cholestatic animals. More importantly, heme was able to enhance urinary BA clearance both in CON and especially in EE-treated animals. Unlike in the liver, heme was able to promote adaptive renal transporter changes by increasing transporters responsible for renal clearance (*Mrp4*, *Mrp2*) and decreasing those for renal BA reabsorption (*Mrp3*) 47. Tissue-specific differences in regulation of renal and liver transporters have been described earlier in obstructive cholestasis 48. In accordance with these data, we have observed a marked reduction in liver *Mrp2* expression with its concomitant slight elevation in the kidney following EE administration and even more pronounced increase after heme pre-treatment. Accordingly, regulation of *Mrp3* expression by heme seems to be tissue-specific as well. While up-regulated in the liver, heme led to a significant decrease in its expression in the kidney (Fig.5).

There are some limitations of our study. To assess the exact contribution of HMOX and/or heme signalling as anti-cholestatic agents, studies with HMOX1 knockout animals should be performed. However, to our knowledge, HMOX1 knockout rats are not available and mice do not develop cholestasis after EE administration and thus cannot be used for this type of experiments. Secondly, further studies should be performed to clarify the mechanism of heme-induced Mrp3 downregulation in rat kidney and also the putative effects of heme administration on BA synthesis and its enterohepatic circulation. Lastly, studies on different animal species and also clinical studies in human are needed to further confirm the feasibility of this approach to treat estrogen-induced cholestasis in humans.

We conclude that the induction of HMOX1 by heme increases expression of hepatocyte membrane transporters, subsequently stimulating bile flow in cholestatic rats. Moreover, heme stimulates hepatic expression of Mrp3 *via* a Nrf2-dependent mechanism. Conjugated BAs, transported by Mrp3 to plasma, are efficiently cleared by the kidneys resulting in normal plasma BA levels. Thus, the HMOX1 induction might represent a potential therapeutic strategy for the treatment of estrogen-induced cholestasis.
